# Can training non-physician clinicians/associate clinicians (NPCs/ACs) in emergency obstetric, neonatal care and clinical leadership make a difference to practice and help towards reductions in maternal and neonatal mortality in rural Tanzania? The ETATMBA project

**DOI:** 10.1136/bmjopen-2015-008999

**Published:** 2016-02-12

**Authors:** David R Ellard, Aloisia Shemdoe, Festo Mazuguni, Godfrey Mbaruku, David Davies, Paul Kihaile, Senga Pemba, Staffan Bergström, Angelo Nyamtema, Hamed-Mahfoudh Mohamed, Joseph Paul O'Hare

**Affiliations:** 1 Warwick Clinical Trials Unit, Division of Health Sciences, Warwick Medical School, The University of Warwick, Coventry, UK; 2 Ifakara Health Institute, Dar es Salaam, Tanzania; 3 Educational Development & Research Team, Warwick Medical School, The University of Warwick, Coventry, UK; 4 Tanzanian Training Centre for International Health, Ifakara, Tanzania; 5 Department of Public Health Sciences, Karolinska Institutet, Stockholm, Sweden; 6 Division of Metabolic and Vascular Health, Warwick Medical School, The University of Warwick, Coventry, UK

**Keywords:** human resources, maternal mortality, Tanzania, Non-physician clinicians, Associate clinicians, medical education and training

## Abstract

**Objectives:**

During late 2010, 36 trainees including 19 assistant medical officers (AMOs) 1 senior clinical officer (CO) and 16 nurse midwives/nurses were recruited from districts across rural Tanzania and invited to join the Enhancing Human Resources and Use of Appropriate Technologies for Maternal and Perinatal Survival in the sub-Saharan Africa (ETATMBA) training programme. The ETATMBA project was training associate clinicians (ACs) as advanced clinical leaders in emergency obstetric care. The trainees returned to health facilities across the country with the hope of being able to apply their new skills and knowledge. The main aim of this study was to explore the impact of the ETATMBA training on health outcomes including maternal and neonatal morbidity and mortality in their facilities. Secondly, to explore the challenges faced in working in these health facilities.

**Design:**

The study is a pre-examination/postexamination of maternal and neonatal health indicators and a survey of health facilities in rural Tanzania. The facilities surveyed were those in which ETATMBA trainees were placed post-training. The maternal and neonatal indicators were collected for 2011 and 2013 and the survey of the facilities was in early 2014.

**Results:**

16 of 17 facilities were surveyed. Maternal deaths show a non-significant downward trend over the 2 years (282–232 cases/100 000 live births). There were no significant differences in maternal, neonatal and birth complication variables across the time-points. The survey of facilities revealed shortages in key areas and some are a serious concern.

**Conclusions:**

This study represents a snapshot of rural health facilities providing maternal and neonatal care in Tanzania. Enhancing knowledge, practical skills, and clinical leadership of ACs may have a positive impact on health outcomes. However, any impact may be confounded by the significant challenges in delivering a service in terms of resources. Thus, training may be beneficial, but it requires an infrastructure that supports it.

**Strengths and limitations of this study**

- This is one of the first studies taking an in-depth look at the impact on health outcomes in districts across rural Tanzania, of a programme of knowledge, skills and clinical leadership training for associate clinicians.
- This cadre is an important component in helping relieve the chronic shortages of trained medical professionals in sub-Saharan Africa and helping countries move towards realisation of millennium development goals.
- One of the primary outcomes (neonatal mortality) was found to be not recorded or poorly recorded at health facilities at the time, preventing us from reporting on this important outcome.
- This was a before and after design and there was no control group with which to draw comparisons.
- A number of facilities where trainees were returned to post-training were not upgraded, as planned, thus preventing them from putting into practice their new skills and knowledge.

## Background

In 2013, it was estimated that there was a global shortage of 7.2 million healthcare workers, and that by 2035 this is expected to rise to 12.9 million.^1^ A recent review of global surgery, obstetric and anaesthesia workforce literature highlights the crisis. Countries such as Tanzania have a physician density of only 1/100 000 people.^2^ It is estimated that currently there is a shortage of 1 million healthcare workers in sub-Saharan Africa.^3^ This shortage is partly because not enough people are appropriately trained but is aggravated by meagre salaries, poor working conditions, low morale, inadequate remuneration, and few opportunities for continuous professional development.^4^ Even with a proliferation of new medical and nursing schools in recent years, the rise is not proportional to the existing large populations.^5^ For those working in rural areas, there is professional isolation, inadequate communication with peers and consultants in the cities, and a lack of appropriate equipment and technologies.^3^

In Tanzania, the lack of basic items in many health facilities has hindered timely and appropriate quality obstetric and neonatal care, particularly in rural and remote health facilities. A number of studies conducted in the country have also indicated that poor quality of care has been experienced at health facilities due to the lack of an enabling environment (drugs, equipment and supplies),^6^ poor skills of providers or hostile attitudes of providers, and a lack of trained staff.^7–10^ As part of the solution, many African countries have created a cadre of mid-level health workers called non physician clinicians (NPCs), now more usually referred to as associate clinicians (ACs). In Tanzania, this cadre is often referred to as clinical officers (COs) or assistant medical officers (AMOs) (COs who have received some additional training). These workers are trained by both government and non-government institutions and are often the most experienced health workers in hospitals and health centres across the country, particularly away from urban centres.^11^ Moreover, all of these AMOs/COs are trained in emergency obstetric care (EmOC) and are in the front line providing care for mothers and babies.^12^ In rural areas where medical doctors (MDs) are few in number, the use of AMOs/COs and nurse midwives (NMWs) has been identified as a viable solution, as these groups can be trained through short course programmes to provide effective comprehensive EmOC (CEmOC) services in remote health centres. The key benefits of using AMOs/COs in CEmOC services include: reducing training and employment costs, promoting task sharing/shifting and enhancing retention within local health systems. Studies have shown that unlike MDs, AMOs/COs remain in rural areas and continue working there.^13^

Major surveys consistently show that additional training and support can enhance task sharing/shifting and reduce maternal and neonatal mortality and morbidity in the areas where these schemes have been piloted.^12^ ^14^ ^15^ Training skilled attendants to prevent, detect and manage major obstetric complications including undertaking emergency caesarean surgery in complicated deliveries is arguably the single most important factor in preventing maternal deaths and protecting the human rights of women.^12^ ^14–16^ To be effective, AMOs/COs need appropriate knowledge, skills, equipment, drugs and technology essential for managing obstetric complications in rural or deprived communities.

The aim of the Enhancing Human Resources and Use of Appropriate Technologies for Maternal and Perinatal Survival in sub-Saharan Africa (ETATMBA) project was to develop, implement and evaluate a programme of locally based clinical service improvement including clinical guidelines and pathways, workforce development through structured education, and leadership training.^17^ ^18^ This was linked to specialist on-site support and mentoring.

## The ETATMBA project in Tanzania

The ETATMBA project combined two main interventions: first, the training of ACs and nurses in CEmOC and anaesthesia. Second, post-training mentoring and supervision of participants at their working places. Within this project, the clinical service improvement involved implementing best existing practice, linked to training in clinical leadership, and providing the context for understanding the additional health gain from the use of appropriate available technologies designed to reduce morbidity-specific maternal case fatality rates and fresh stillbirth rates (intrapartum fetal mortality) across different African communities (Malawi and Tanzania).^19^ See also web supplementary material for additional information.

The main aim of this study was to explore the impact of the ETATMBA training on health outcomes including maternal and neonatal morbidity and mortality in the facilities where trainees were based. Secondly, surveying these health facilities and looking at their ability to support the trainees in terms of infrastructure, supplies and drugs. We were exploring the facilitators and barriers to good clinical practice and the day-to-day challenges faced by the health workers in these facilities. In addition, a qualitative study was undertaken time with the trainees and other stakeholders, but this will be reported elsewhere.^20^

## Methods

### Design

The study is a pre-examination and postexamination of maternal and neonatal health indicators and a survey of a sample of health facilities in rural Tanzania. The survey includes: infrastructure, availability of equipment, supplies and drugs. The facilities surveyed were those in which ETATMBA trainees were placed post-training. The health indicators were collected for the whole of 2011 (pre) and the whole of 2013 (post): the survey of the facilities was in early 2014.

### Participants

During late 2010 and 2011, 36 trainees (AMOs and NMWs/nurses (anaesthesia)) were recruited from districts across Tanzania and invited to undertake the ETATMBA training programme (see web appendix for more information).

### Outcome measure

Maternal and neonatal health outcomes were collected from each health facility where a trainee was based (post-training) for the whole of 2011 (pre) and 2013 (post). This included early neonatal mortality (only including deaths that occur before discharge) and maternal mortality (case-specific) and other obstetric indicators including: numbers of birth events, stillbirths, postpartum haemorrhage, caesarean sections, obstructed labour and sepsis. It is important to note here that neonatal mortality rates were not reported in the baseline data, we believed they had been overlooked. We planned to rectify this by retrospectively collecting the data. However, after we visited the sites, it became clear that neonatal mortality rates had not been recorded, or at least were not available. These data were therefore unavailable for either baseline or follow-up.

The outcomes selected all relate to ETATMBA knowledge and skills training, and rely on data believed to be available in monthly/annual summary reports stored at each facility covering the project time period. A 10% sample of variables was cross-checked with the actual registers for accuracy at each facility.

A predesigned instrument was used to capture the survey data (see online supplementary appendix 1). This captured the availability of resources including equipment, supplies and infrastructure, and recorded whether there was a sufficient supply/number of the listed items for the facility's daily caseload of deliveries, and whether the items had been available and functional, available but not functional, or not available (eg, infrastructure, equipment, supplies and drugs). Essential drugs: the availability and supply of drugs for each room (emergency room, labour/delivery room, maternity ward, operating theatre and pharmacy) were recorded. Checks were done to confirm whether the listed drug was available and if the supply was sufficient to last for <1 week, up to 1 week, up to 2 weeks, up to 3 weeks, or up to 4 or more weeks.

### Research team

The primary data collection team consisted of two local research assistants based at the Ifakara Health Institute (IHI), Dar es Salaam, Tanzania. Both of the research assistants are experienced researchers. The principal investigator at the IHI gave local support, with management/oversight provided by DRE at Warwick.

### Procedure

The research assistants identified the facilities in which trainees were working and extracted the 2011 study variables from data collected by colleagues at IHI for ETATMBA in 2012 (baseline data). The follow-up data were the same variables for the year 2013. The follow-up data and the facility survey data were gathered during site visits to the facilities in early 2014.

## Data analysis

Descriptive and summary statistics were produced for the 2 years, change scores were produced, and Student t tests were used to look for differences. Significance was set at 5%. Data are presented in tables and graphs as appropriate. Survey data are presented as descriptive statistics. Data are grouped by facility type (ie, district hospitals and health centres) as they are different. In simple terms, we expected the hospitals to be larger than health centres and have more staff and better availability of essential infrastructure, supplies, equipment and drugs.

## Results

Post-training, ETATMBA trainees returned to 17 rural health facilities in Tanzania. Sixteen of these health facilities were included in this study. Table 1 gives an overview of the facilities and the ETATMBA trainees who were based there after the training. Thirty-six received the ETATMBA training including 19 AMOs, 1 CO and 14 NMWs and 2 nurses (anaesthesia). During the project period, one AMO and one NMW left the programme to pursue other interests and one NMW died. Thus, attrition at the end of the training programme was around 8%. Fourteen trainees did not return to the facility from which they were recruited because the facilities had not received an expected facility upgrade.

**Table 1:** Health facilities where the Tanzanian ETATMBA trainees were based in 2013

	District	Name of facility	Operating theatre	CEmOC or BEmOC	Number of trainees
1	Bukombe	Bukombe District Hospital	Yes	CEmOC	1 AMO
2	Bukombe	Uyovu Health Centre	No	BEmOC	1 AMO, 1 CO
3	Geita	Nzela Health Centre	Yes	CEmOC	1 NMW, 1 Nurse
4	Geita	Katoro Health Centre	No	BEmOC	1 AMO, 1 NMW
5	Inyonga	Mamba Health Centre	Yes	CEmOC	1 NMW
6	Karambo	Matai Health Centre	No	BEmOC	1 AMO, 1 NMW
7	Liwale	Liwale District Hospital	No	CEmOC	2 AMOs
8	Meatu	Mwandoya Health Centre	No	BEmOC	1 AMO, 1 NMW
9	Mpanda	Mpanda District Hospital	Yes	BEmOC	1 AMO, 1 Nurse
10	Nachingwea	Nachingwea District Hospital	Yes	CEmOC	2 AMOs
11	Nkasi	Kirando Health Centre	Yes	CEmOC	2 AMOs
12	Nyanghwale	Nyanghwale Health Centre	No	BEmOC	1 AMO, 1 NMW
13	Nyanghwale	Kharumwa District Hospital*	Yes	CEmOC	1 AMO, 1 NMW
14	Ruangwa	Ruangwa District Hospital	Yes	CEmOC	1 AMO, 1 NMW
15	Sumbawanga	Laela Health Centre	No	BEmOC	1 AMO, 1 NMW
16	Chato	Chato District Hospital	Yes	CEmOC	1 AMO, 1 NMW
17	Lindi	Nyangao Mission Hospital†	Unknown	CEmOC	2 NMWs

*Upgraded to a district hospital between 2011 and 2013.

†This hospital was not visited, so it is not included in the analysis.

AMO, assistant medical officer; BEmOC, basic emergency obstetric care; CEmOC, comprehensive emergency obstetric care; CO, clinical officer; ETATMBA, Enhancing Human Resources and Use of Appropriate Technologies for Maternal and Perinatal Survival in the sub-Saharan Africa; NMW, nurse midwife; Nurse, nurse/anaesthetics.

Box 1 provides a United Nations definition of basic and comprehensive emergency obstetric and newborn care (BEmOC and CEmOC).

Box 1 **Defining basic and comprehensive emergency obstetric and newborn care (BEmOC and CEmOC)**

BEmOC is critical to reducing maternal and neonatal death. This care, which can be provided with skilled staff in health centres, large or small, includes the capabilities for

- Administering antibiotics, uterotonic drugs (oxytocin) and anticonvulsants (magnesium sulfate)
- Manual removal of the placenta
- Removal of retained products following miscarriage or abortion
- Assisted vaginal delivery, preferably with a vacuum extractor
- Basic neonatal resuscitation care

CEmOC, typically delivered in hospitals, includes all the basic functions above, plus capabilities for

- Performing caesarean sections
- Safe blood transfusion
- Provision of care to sick and low birthweight newborns, including resuscitation

Adapted from the United Nations Population Fund material. For more information see: http://www.unfpa.org/resources/setting-standards-emergency-obstetric-and-newborn-care#sthash.5rcjLhLA.dpuf

Table 2 summarises the key obstetric indicator figures from the 16 health facilities for 2011 and 2013.

**Table 2:** Comparison of key maternal, neonatal and birth complication figures from baseline (2011) to follow-up (2013)

	2011			2013			Difference^b−a^*
	DH (n=7)	HC (n=9)	Total^a^	DH (n=7)	HC (n=9)	Total^b^	
Total deliveries	17 893	7326	25 219	16 654	7961	24 615	−604
FSB (n)	287	65	352	300	68	368	16.0
FSB rate (per 1000 births)	16.0	8.9	14.0	18.0	8.5	15.0	1.0
MSB (n)	312	61	373	305	111	416	43.0
MSB rate (per 1000 births)	17.4	8.3	14.8	18.3	13.9	16.9	2.1
Maternal deaths (n)	68	3	71	55	2	57	−14.0
MD ratio (per 100 000 births)	380	41	282	330	25	232	−50
CS deliveries (n)	1944	78	2022	1851	49	1900	−122
CS rate (per 1000 births)	108.6	10.6	80.2	111.1	6.2	77.2	−3.0
PPH (n)	200	77	277	225	86	311	34.0
PPH rate (per 1000 births)	11.2	10.5	11.0	13.5	10.8	12.6	1.7
Obstructed labour (n)	114	49	163	159	23	182	19.0
Obstructed labour rate (per 1000 births)	6.4	6.7	6.5	9.5	2.9	7.4	0.9
Sepsis (n)	31	12	43	51	4	55	12.0
Sepsis rate (per 1000 births)	1.7	1.6	1.7	3.1	0.5	2.2	0.5

*There are no significant differences here, so p values are not shown.

CS, caesarean section; DH, district hospitals; FSB, fresh stillbirth; HC, health centres; MD, medical doctor; MSB, macerated stillbirth; PPH, postpartum haemorrhage.

No significant differences were found for any of the key obstetric variables across the lifetime of the project. The number of deliveries/births decreases slightly overall (−604), but the number of deliveries/birth in health centres rises (from 7326 to 7961). There is only a slight increase in overall fresh stillbirths (+16, an increase of 1 case/1000 births), while there is an increase in macerated stillbirths in health centres (from 8.3 to 13.9 cases/1000 live births). Maternal death ratios show a downward, improving trend over the 2 years (down from 282 to 232 cases/100 000 live births), but this is not statistically significant. There is a reduction in the caesarean section rate overall, down from 80.2 to 77.2 (cases per 1000 live births), with a large reduction in health centres where rates are down from 10.6 to 6.2 (cases per 1000 live births), while in the hospitals there is an increase in the rate from 108.2 to 111.1 (cases per 1000 live births). The birth complication variables collected all show a slight increase overall, but each shows a differing trend in where they were reported. The rates of postpartum haemorrhage change little over time. Obstructed labour rates increased in district hospitals (6.4–9.5 cases/1000 live births), while in health centres there was a decrease (6.7–2.9 cases/1000 live births). Sepsis follows a similar trend with an increase in hospitals (1.7–3.1 cases/1000 live births) and a decrease in health centres (1.6–0.5 cases/1000 live births).

## Facility survey results

These results originate from the survey undertaken in early 2014 by IHI researchers. As noted in table 1 above, there were 17 facilities across the country that housed ETATMBA trainees during this survey. One of these facilities (owing to its distance and remoteness) was not visited. All results are based on 16 facilities, 9 health centres and 7 district hospitals.

### Facilities: overall capacity and infrastructure

Availability of running water and functioning toilets are a very significant problem with only one of nine health centres (11%) and four of seven (57%) district hospitals found to have the availability of running water and only just over half of the facilities a functioning toilet (56%). Most facilities had sufficient access to lighting to perform tasks at night (75%) but clearly there was still some struggle. Delivery beds were found to be available in 56% of the health centres and 86% of the district hospitals. Ambulance availability was poor at health centres with only one (11%) having availability, whereas five of the seven (71%) district hospitals had an ambulance available. Referrals from within the maternity area are problematic as only four health centres had a working (landline) phone in this area and none of the district hospitals had any. The availability of health-related registers/records is variable, varying from 100% for items such as the delivery register and monthly/annual reports to 6% or less for the gynaecology register, patient records and discharge registers (table 3).

**Table 3:** Survey findings from health facilities in Tanzania related to infrastructure

	Facilities with the items		
	Overall (%)	HC (%) n=9	DH (%) n=7
Health facility infrastructure availability of power and availability of water			
Sufficient light source to perform tasks at night	12 (75)	6 (67)	6 (86)
Means of ventilation	5 (31)	1 (11)	4 (57)
Running water	5 (31)	1 (11)	4 (57)
Functioning toilet	9 (56)	6 (67)	3 (43)
Functional fan/air conditioning	5 (31)	1 (11)	4 (57)
Curtains/means of providing patient privacy	14 (88)	9 (100)	5 (71)
Waiting area for visitors and family	6 (38)	4 (43)	2 (33)
Facility with electricity	14 (89)	8 (86)	6 (86)
Ambulance – available and functional	6 (38)	1 (11)	5 (71)
Available and functional landline telephone in the maternity area	4 (25)	4 (43)	0 (0)
Delivery bed/table	11 (69)	5 (56)	6 (86)
Availability of health-related registers			
General admission register	11 (69)	5 (56)	6 (86)
Delivery register	16 (100)	9 (100)	7 (100)
Maternity ward register	9 (56)	4 (44)	5 (71)
Female ward register	9 (56)	4 (44)	5 (71)
Operating theatre register	10 (63)	4 (44)	6 (86)
Gynaecology register	0 (0)	0	0
Postabortion register	9 (56)	4 (44)	5 (71)
Individual patient records	1 (6)	0	1 (14)
Discharge register	1 (6)	0	1 (14)
Death register	11 (69)	6 (67)	5 (71)
Mortuary register	7 (44)	2 (22)	5 (71)
Monthly/annual facility summary reports	16 (100)	9 (100)	7 (100)

DH, district hospitals; HC, health centres.

### Drugs and equipment for normal delivery and infection prevention

Generally, supplies and equipment availability were at an acceptable level but there are a number of exceptions. Only about 50% of facilities had needles and syringes available, and similarly availability of suction and vacuum extraction equipment was low. The availability of drugs for normal delivery purposes was very variable with some drugs readily available (eg, lignocaine), while others had very low stocks (eg, injectable antibiotic and diazepam) (table 4).

**Table 4:** Survey findings from health facilities in Tanzania related to the availability of equipment, supplies and drugs

	Facilities with the equipment		
	Overall (%) N=16	HC (%) N=9	DH (%) N=7
*Drugs and equipment: availability of items for normal delivery*			
Equipment and supplies			
Blood pressure cuff/machine	13 (81)	7 (78)	6 (86)
Stethoscope	15 (94)	8 (89)	7 (100)
Fetal stethoscope	16 (100)	9 (100)	7 (100)
Clinical thermometer	13 (81)	6 (67)	7 (100)
Sterile gloves	16 (100)	9 (100)	7 (100)
Non-sterile protective clothing/apron	15 (94)	8 (89)	7 (100)
Scissors or razor blade for cutting cord	15 (94)	9 (100)	6 (86)
Cord ties	10 (63)	5 (56)	5 (71)
Needles and syringes	8 (50)	4 (44)	4 (57)
Intravenous fluid set (giving set)	15 (94)	9 (100)	6 (86)
Suture needles and suture materials	10 (63)	5 (56)	5 (71)
Suction apparatus	8 (50)	3 (33)	5 (71)
Manual vacuum extractor	5 (31)	2 (33)	2 (29)
Obstetric forceps	11 (69)	8 (89)	3 (43)
Drugs			
Pitocin (oxytocin)	13 (81)	6 (67)	7 (100)
Ergometrine (injectable)	4 (25)	3 (33)	1 (14)
Normal saline	14 (88)	8 (89)	6 (86)
Ringer's lactate	7 (44)	2 (22)	5 (71)
Dextrose/glucose	9 (56)	3 (33)	6 (86)
Lignocaine 2% or 1%	15 (94)	8 (89)	7 (100)
Injectable antibiotic	5 (31)	3 (33)	2 (29)
Magnesium sulfate	14 (88)	8 (89)	6 (86)
Diazepam	6 (38)	3 (33)	3 (43)
Skin disinfectant	12 (75)	7 (78)	5 (71)
*Availability of infection prevention services in labour delivery/operating theatres*			
Decontamination container with prepared solution	11 (69)	5 (56)	6 (86)
Covered contaminated trash bin	11 (69)	6 (67)	5 (71)
Sharps disposal container	12 (75)	6 (67)	6 (86)
Soap	0	0	0
Antiseptics	10 (63)	5 (56)	5 (71)
Chlorine/bleach	6 (38)	2 (22)	4 (57)
Sterile gloves	12 (75)	6 (67)	6 (86)
Other items			
Regular trash bin	12 (75)	6 (67)	6 (86)
Non-sterile gloves	12 (75)	6 (67)	6 (86)
Non-sterile protective clothing	12 (75)	6 (67)	6 (86)

DH, district hospitals; HC, health centres.

### Infection prevention services in labour delivery/operating theatres

Overall, only 75% or less of the facilities surveyed had the basics for infection prevention. None seemed to have regular availability of soap for hand washing, although antiseptics and bleach were available and may be used as alternatives (table 4).

### Comprehensive services for provision of anaesthesia

Most of the district hospitals surveyed had good availability of equipment and supplies for anaesthesia, although Halothane is only available in 3/7 district hospitals and <40% overall. Health centres seemed to lack access to oxygen with only 2/9 having supplies when surveyed (table 5).

**Table 5:** Survey findings from health facilities in Tanzania related to the availability of items for management of anaesthesia, birth complications and caesarean section

Equipment	Facilities with the items		
	Overall N=16 (%)	HC N=9 (%)	DH N=7 (%)
Items for provision of anaesthesia			
Suction machine	6 (38)	4 (44)	2 (29)
Filled oxygen cylinder with cylinder carrier and key to open valve	8 (50)	2 (22)	6 (86)
Intubating forceps (Magill)	6 (38)	4 (44)	2 (29)
Adult laryngoscope	11 (69)	6 (67)	5 (71)
Adult ventilator bag and mask	11 (69)	6 (67)	5 (71)
Intravenous fluid set (giving set)	10 (63)	5 (56)	5 (71)
Spinal needles (18–25 gauge)	3 (19)	1 (11)	2 (29)
Endotracheal tubes with cuffs (8–10 mm)	9 (56)	4 (44)	5 (71)
Halothane	6 (38)	3 (33)	3 (43)
Ketamine	11 (6)	5 (56)	6 (86)
Anaesthetic face masks	9 (56)	5 (56)	4 (57)
Items for management of pre-eclampsia/eclampsia			
Magnesium sulfate	7 (44)	4 (44)	3 (43)
Diazepam (injectable)	10 (63)	4 (44)	6 (86)
Nifedipine	1 (6)	0 (0)	1 (14)
Blood pressure cuff/machine	13 (81)	7 (78)	6 (86)
Stethoscope	15 (94)	8 (89)	7 (100)
Adult ventilator bag and mask	13 (81)	7 (78)	6 (86)
Needles and syringes	4 (25)	1 (11)	3 (43)
Urinary catheters (Foleys)	8 (50)	3 (33)	5 (71)
Uristix	4 (25)	1 (11)	3 (43)
Items for management of haemorrhage (parenteral uterotonics)			
Needles and syringes	8 (50)	4 (44)	4 (57)
Intravenous fluid set (giving set)	9 (56)	3 (33)	6 (86)
Items for caesarean section (not including anaesthesia)			
Operating table			
Light-adjustable, shadowless	11 (69)	6 (56)	5 (86)
Antiseptics	10 (63)	5 (56)	5 (71)
Sterile gloves	12 (75)	6 (67)	6 (86)
Cord ties	10 (63)	5 (56)	5 (71)
Needles and syringes	6 (38)	4 (44)	2 (29)
Benzyl penicillin	4 (25)	3 (33)	1 (14)
Metronidazole (intravenously)	2 (13)	1 (11)	1 (14)
Gentamycin (intravenously)	1 (6)	1 (11)	0 (0)
Caesarean section pack			
Needle holder	13 (81)	7 (78)	6 (86)
Scalpel handle with blade	10 (63)	5 (56)	5 (71)
Retractor	12 (75)	6 (67)	6 (86)
Surgical scissors	12 (75)	6 (67)	6 (86)
Suction apparatus/8*	6 (38)	4 (44)	2 (29)
Oxygen	8 (50)	2 (22)	6 (86)
Sutures	11 (69)	5 (56)	6 (86)
Ketamine	11 (69)	5 (56)	6 (86)
Lidocaine/5*	12 (75)	6 (67)	6 (86)

*The numbers against these items (8 and 5) are the units for this item to be classed as available (eg, there has to be 8 suction apparatus for it to be classed as available).

DH, district hospitals; HC, health centres.

### Items for management of birth complications and caesarean section

Overall, unsurprisingly, district hospitals had better availability of equipment, drugs and supplies for managing birth complications and for performing caesarean sections (table 5).

## Discussion

The main objective of this study was to explore the impact of the ETATMBA training on health outcomes including maternal and neonatal morbidity and mortality in the facilities where trainees were based. Secondly, surveying these health facilities and looking at their ability to support the trainees in terms of infrastructure, supplies and drugs. We were looking for facilitators and barriers to good clinical practice and the day-to-day challenges faced by the health workers in these facilities. We were successful in collecting data for the pre and post comparisons and also the survey data.

Interestingly, our study shows that the number of actual births decreased overall in the 16 facilities measured between 2011 and 2013. The reduction was seen mostly at the district hospitals with numbers increasing at health centres. There was a slight increase in fresh stillbirths, but again most of this is at the district hospitals rather than at the health centres. This may suggest that health centres are referring more women with this problem, but the number of macerated stillbirths increased both in district hospitals and health centres, with the latter showing the bigger rise. This trend should be interpreted with caution, since distinction of the type of stillbirth is known to be variable in quality, and indeed it may just suggest that women are presenting late at the health facilities.

Maternal deaths decreased, which is encouraging as this was a goal of the ETATMBA training. However, it is not a statistically significant reduction, rather a downward trend. This could simply be a reflection of the reduction in maternal mortality reported in recent years across Tanzania.

Neonatal mortality was one of our key health indicators in this study. However, it was found that neonatal mortality was not recorded on the Ministry of Health monthly summary sheets in facilities and thus was not available to us. Reducing neonatal mortality is one of the WHO millennium development goals.^21^ While the number of stillbirths was routinely recorded, early neonatal deaths were not. This was a very disappointing outcome as a key component of the ETATMBA training was aimed at interventions to prevent neonatal deaths (ie, deaths at or around the time of birth and before discharge from hospital).^19^ Indeed, in Malawi, we have very positive indications that the ETATMBA training has helped to save neonate lives.^22^ Our study has acted as a ‘wake-up call’ to the Ministry of Health and Social Welfare (MoHSW) in Tanzania, who have now updated the current Health Management Information System (HIMS) to ensure that neonatal data are collected.

Looking at the birth complication data (postpartum haemorrhage, obstructed labour and sepsis), all are seen to rise from 2011 to 2013 both in district hospitals and health centres with one exception. Sepsis rates in health centres decreased, though great caution must be observed, since registration of morbidities is often incomplete and the facility survey showed centres lacking basic hygiene resources such as soap. It is a matter of some concern that the number of complications is increasing, but this could be a reflection of more women getting to a health facility where there are health staff who can deal with the problems. Despite the increase in the numbers of mothers with obstructed labour and postpartum haemorrhage, it is encouraging that maternal mortality ratios at these facilities appear to be falling. The observed incidence increase in these two registered morbidities in all probability implies an enhanced recognition and registration of them, rather than a higher incidence in the facility population under study. We do need to be cautious in our interpretation of these data with only before and after data, as there is no control to detect temporal trends that may be occurring across Tanzania.

While our focus in this study was on the facilities where ETATMBA trainees returned to after their training, it is important to draw attention to events that were outside the control of the ETATMBA team, events that may have influenced the outcomes. Prior to recruitment, the ETATMBA trainees were based in health centres and district hospitals across rural Tanzania. The original MoHSW plan was to recruit trainees from health facilities that were due to be upgraded with a theatre and maternity ward including equipment and resources so that trainees could implement their new skills. However, the reality was that of the 33 trainees who completed the programme, only 19 returned to the place from where they were selected and 7 of these returned to facilities that had not been upgraded or where upgrading was still in process. Fourteen trainees did not return to the facility from which they were recruited because the facilities had not been upgraded. Of these, 10/14 were returned to district hospitals in the area they had originally come from. Often, these decisions were made by local District Medical Officers pragmatically responding to need and not to the strategic planning of the MoHSW. Upgrading of facilities was not part of the ETATMBA project but rather was ongoing work with the Tanzanian Government and other funding agencies. It is clear that in a number of cases trainees would have struggled to put their new-found skills into practice as facilities they returned to were not conducive to good clinical practice. For those who returned to a district hospital, it could have been a double-edged sword. On the one hand, a district hospital could give many more opportunities to put their new-found skills into practice, but on the other hand the current senior staff may have been reluctant to allow them to practise.

The survey reveals some alarming trends in the availability of resources in these facilities. A facility was designated as a CEmOC where there was no functioning operating theatre and a District hospital was designated as a BEmOC rather than a CEmOC when we conducted a survey. The latter clearly did not meet all the requirements for a CEmOC at the time of the survey. There are considerable shortages in basic infrastructure such as running water, electricity and toilet facilities. The Survey asks for a land line telephone to available as standard most did not have this. However, mobile phones are being used more and more now in Africa and are more reliable in terms of service provision. Future surveys should take this into account.

Record keeping in the facilities is also very variable. Monthly/annual summary reports (containing the data we required) were available in all facilities and in most we were able to cross-check the data with register records, but some registers were missing and we have already noted the issues surrounding neonatal mortality rates.

The survey reveals shortages in equipment, supplies and drugs that could impact on patient care. The district hospitals are better supplied than the health centres. This may be due to the remoteness of the health centres, but there are disturbing shortages of the basics for infection/hygiene control and the provision of oxygen. Infection prevention services were extremely poor. Basic items like soap for hand washing were mostly absent. However, sepsis rates, although rising slightly overall, were not significantly different to baseline (2011) levels, suggesting that despite enormous challenges and a lack of even basic supplies and equipment, these clinicians managed to contain sepsis in their facilities. Our survey findings are all the more alarming as they seem to mirror a more comprehensive survey performed back in 2005/2006, suggesting that things have not changed a great deal.^6^ Despite all of this, it does seem that in the face of all of these challenges things are not getting any worse but they could be better.

This study has a number of limitations, not least that one of the primary outcomes was not available to us. The sample is small and may not be representative of all facilities across Tanzania, with generally only two trainees in each facility with large throughputs of cases/births. We are not comparing our facilities to control districts, so it is difficult to attribute changes just to ETATMBA training. Another limiting factor is that ETATMBA had no control over where trainees returned to post-training, and a significant number returned to facilities where they could not practise their new-found skills. This, however, did mean that our sample was more random (not chosen by us). Finally, this project needs to be seen in the context of the vast distances between facilities and how the terrain and weather impacts on the health service provision in rural Tanzania. Indeed, in 2009, Evjen-Olsen *et al*^23^ suggest the need for an integrated and comprehensive hospital-based/community-based approach to obstetric healthcare in rural Tanzania, but our experience here has not shown this being put into practice.

Earlier findings from this project suggested that the training had an impact, at the local level, on maternal mortality.^24^ Sadly, in this larger current study, we cannot be certain of this conclusion although our qualitative findings provide some corroboration to our positive findings.^20^ It is acknowledged that maternal mortality is still a significant problem, particularly in rural Tanzania.^25^ Nelissen *et al*^25^ suggest that there is a great need for the upscaling and use of evidence-based interventions that could help to save lives. We can only hope that the ETATMBA training, which is grounded in evidence based medicine, and its trainees will be a stimulus to improved care. However, for a full impact, the implementation of the training needs to be linked to the provision of well-supplied healthcare facilities in the remote areas. We note that in one province the ETATMBA training has influenced the upgrade of more health centres at the district level in tandem with the MoHSW objective of upgrading at least 50% of all health centres in a particular province to provide CEmOC.^26^

A number of papers still highlight that women are reluctant to attend rural health facilities as they believe the standard of care they will receive will be poor and many still give birth at home without skilled birth attendance.^9^ ^27^ We can only hope that the upskilling of health providers in these rural areas cascades within the communities to encourage women to seek skilled help during birth.

While not a direct result of our work during the lifetime of this project, there has been a shift in acknowledging the importance of this cadre of health workers. The negative label NPC has been replaced with the more dignifying and respectful AC. ACs are now coming together across Africa starting their own professional association. Indeed, there is now a very active network called African Network of Associate Clinicians (ANAC) enabling the formation of a community of practice.

Comparing our results with those from Malawi in this project, we see an indication that the ETATMBA training can make a difference.^22^ There are similarities and differences between this study and that carried out in Malawi, but in both countries it seems that overall the outcomes have been very positive.

We know that the ETATMBA training was successfully implemented (we were able to train the ACs and we know we have improved their leadership, knowledge and clinical skills), but we are still unclear about the impact in Tanzania. We interpret our results here with caution, presenting just exactly what we found. There are trends in the data, which suggest an improving picture. However, it seems that the full impact of the training at a community level does not as yet show in the results. We believe that the dedication shown by the trainees, coupled with their new skills and knowledge, will have a positive impact over the coming years as more health centres are upgraded and adequately resourced.
